# The effectiveness of marine protected areas on the spatio-temporal patterns of reef fish in the Southwest Atlantic

**DOI:** 10.1098/rsos.241092

**Published:** 2025-04-09

**Authors:** Amanda Aparecida Carminatto, Paulo Emilio Costa Santos, Rodrigo de Oliveira Campos, Matheus Marcos Rotundo, Davi Butturi-Gomes, Miguel Petrere Jr.

**Affiliations:** ^1^ Graduate Program in Coastal and Marine Ecosystem Sustainability, Universidade Santa Cecília, Santos, Boqueirão, Brazil; ^2^ Graduate Program in Planning and Use of Renewable Resources, Universidade Federal de São Carlos, Sorocaba, Bairro do Itinga, Brazil; ^3^ Amazon Aquatic Technology and Fisheries Center, Universidade Federal do Pará, Belem, Guamá, Brazil; ^4^ Graduate Program in Environmental Science and Technology, Universidade Santa Cecília, Santos, Boqueirão, Brazil; ^5^ Graduate Programs in Environmental Auditing, Universidade Santa Cecília, Santos, Boqueirão, Brazil; ^6^ DEMAT – Departamento de Matemática e Estatística, Universidade Federal de São João del-Rei, Sao Joao del-Rei, Minas Gerais, Brazil; ^7^ Graduate Program in Coastal and Marine Ecosystem Sustainability, Universidade Santa Cecilia, Santos, São Paulo, Brazil

**Keywords:** reef fish ecology, marine protected areas, remotely operated vehicle, seasonal variation, diurnal variation, conservation target species, fishing target species, schooling species

## Abstract

This study explored spatio-temporal patterns influencing reef fish richness and abundance in two coastal islands within marine protected areas (MPAs) in southeastern Brazil. Data were collected using a remotely operated vehicle (ROV) during the 2022 seasonal cycle, with samples taken day and night. A total of 16 661 individuals from 81 species was recorded. The results showed that fish abundance was higher during the day, in winter and in areas with high temperatures. At night, in autumn, behavioural changes were observed in schooling species. Seasonal variations, including temperature changes and the oceanographic characteristics of the study area, influenced fish abundance and species composition, favouring tropical and subtropical species. Depth affected the islands differently: higher abundance was observed in deeper waters at Anchieta Island State Park, while shallower waters at Mar Virado Island showed greater abundance due to habitat complexity. Diurnal variation in richness was significant at Mar Virado Island, probably due to increased nocturnal predation. For fishing-targeted species, the islands showed significant effects on species composition and abundance, highlighting the importance of protected areas. The study offers key insights into reef fish dynamics, emphasizing the role of spatio-temporal variables in shaping communities and supporting conservation strategies in MPAs.

## 1. Introduction

In recent years, several studies have explored the effect of fishing ban in marine protected areas (MPAs) as an anthropogenic factor influencing fish communities [1–5]. These studies have found that, in general, the abundance, biomass and average size of exploited fish populations are higher within no-take zones than in open areas, both in Brazil [3,4,6] and elsewhere in the world [2,7]. However, research on temporal patterns in fish communities, as well as other organisms, is limited to a single season (mostly in the summer) and in daytime due to the collection of ichthyofauna and/or visual censuses being comparatively easy [1,8,9].

Seasonal variations in reef fish communities may be associated with migration patterns of anadromous species, and the presence of transient marine species, which, in turn, is linked to reproductive movements, shifts in precipitation patterns and recruitment peaks driven by resource availability at specific times of the year [10,11]. Diurnal variations in reef fish communities are associated with ecological factors, such as balance between foraging and predation, habitat use, light availability and temperature [12]. These differences probably result from changes in trophic relationships, including the vertical distribution of prey and behavioural adaptations in response to increased predation during the night time [9,13,14]. Therefore, considering seasonal and diurnal variations is crucial for understanding the dynamics and occurrence of reef fish, especially in MPAs. These variations influence fish behaviour, population dynamics and habitat use, which are essential for effective conservation and management strategies [9,15,16].

Additionally, local-scale diversity has been traditionally explored through richness, abundance and taxonomic composition [17]. Although these metrics have been important in elucidating population trends in abundance and providing essential information about communities, they do not fully reflect the differences among species and their contributions to ecosystem functioning [18,19]. Therefore, the inclusion of metrics associated with behaviour (e.g. schooling or non-schooling species) and their conservation or commercial importance (e.g. species threatened to some degree of extinction and of interest for fishing) are complementary to identifying diversity patterns and ecosystem functioning [20,21], especially in MPAs. This also enables the estimation of reference parameters for conservation and fisheries management.

In Brazil, MPAs are strategically positioned to address future changes in reef environments [22]. However, current management plans require improvements to tackle threats, especially those related to fishing [22,23]. The effectiveness of MPAs depends on a multi-level governmental commitment (municipal, state and federal) to oversee and regulate fishing activities, as well as to promote a shared and effective management among the various users of these areas [22–24]. A survey of reef fish fishing in Brazil from 1950 to 2015 identified a shift in the species caught, with an increasing focus on smaller fish and lower trophic levels [25]. This pattern suggests unsustainable pressure on fishery resources, compounded by the lack of updated official data since 2015, which represents a critical challenge for the management and regulation of fishing activities [25].

There are still knowledge gaps impacting monitoring demands in MPAs in Brazil. Among these demands, those of the northern coastal marine environmental protection area—which encompasses our study areas—stand out, including the investigation of the ichthyofauna of coastal islands and surrounding areas, targeted monitoring of reef species and conducting studies covering various aspects of ichthyofauna, including species behaviour and distribution [26]. In this context, our study aims to acquire and disseminate scientific knowledge, serving as a tool to support the management of these areas and providing essential information for decision-making and the implementation of conservation and preservation actions.

Aiming to expand knowledge on the temporal dynamics of reef fish communities and to support the development of more effective conservation strategies for MPAs in Brazil, we adopted a non-destructive method that complements traditional visual sampling. The use of non-invasive techniques, such as remotely operated vehicles (ROVs), baited remote underwater video (BRUV), remote underwater videos (RUV) and submersible rotating videos (SRV), has proven to be an effective tool for obtaining accurate data on fish communities and is widely used in marine ecosystems, especially in MPAs [4,27–30]. Although the use of ROVs in Brazil is still limited, studies such as those by Pereira-Filho *et al.* [31] have highlighted their application in the analysis of reef fish and benthic habitats in the Trindade and Martin Vaz islands. Silva [32] and Colares [33] utilized ROVs to characterize habitats in MPAs and lobster fishing grounds, demonstrating their importance in the conservation and management of marine resources.

More recently, Carminatto *et al.* [29] pioneered the assessment of seasonal variations in reef fish communities using ROVs in Brazil. However, this is the first study to incorporate, in addition to seasonal variation, diurnal variation in MPAs using an ROV, thereby enhancing our understanding of the temporal dynamics of these communities. Therefore, aiming to understand the spatial–temporal patterns and processes of richness and numerical abundance of rocky reef fish on two coastal islands within an MPA, this paper addresses the following questions: (i) Do numerical abundance and richness vary with spatial predictors (islands), temporal factors (diurnal and seasonal variation) and abiotic factors (temperature and depth)? (ii) Considering schooling and non-schooling species, does the pattern of numerical abundance distribution remain the same with spatial, temporal and abiotic predictors? And (iii) Considering that one of the islands has fishing restrictions, does the composition and proportion of co-occurrences between conservation target species and fishing target species differ between them?

## 2. Material and methods

### 2.1. Study area

**Figure 1.**
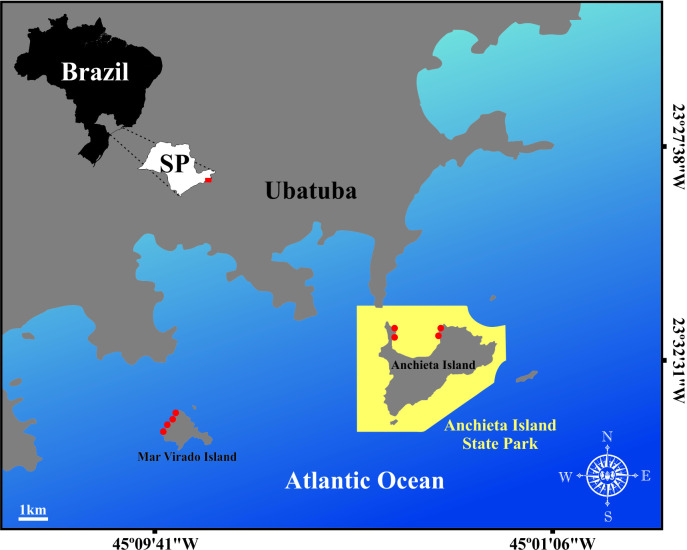
Study areas located in Ubatuba, northern coast of São Paulo, Brazil: Mar Virado Island and Anchieta Island State Park. The red points denote the sampling sites on each island.

The field study was conducted on two coastal islands in Ubatuba, São Paulo, Brazil: the Anchieta Island State Park (PEIA) (23°27’33.04” S, 45°02’45.04” W) and Mar Virado Island (IMV) (23°33’58.98” S, 45°09’25.62” W). PEIA is a full protection conservation unit, established in 1977, with a fishing ban area of 1713 ha since 1983 [34,35]. The island is separated from the mainland by a narrow (0.5 km) and relatively deep (33 m) channel. Its coastline is predominantly granitic, except for a 1.5 km sandy beach on the north side, which is shallower and less exposed. Depths around the island reach up to 37 m, with a benthic mosaic that includes sandy, gravel and rocky bottoms [36]. The IMV has been part of the marine environmental protection area of the northern coast of São Paulo since 2008, allowing fishing methods, such as hooks, spearfishing and gillnets, but prohibiting industrial trawling over 20 gross tonnage [37]. Depths around the island reach up to 14 m, with a granitic coastline and a bottom characterized by rocky formations, located 2 km from the mainland [38] (figure 1).

The region’s climate is classified as humid tropical, with an average annual temperature of 19.5°C in winter and 25.0°C in summer. Temperatures range from a minimum of 3.7°C to a maximum of 40.8°C. The average annual relative humidity is 84.8%, and the average annual precipitation is 1818 mm, with the summer months showing the highest rainfall levels, while winter is the driest period [36,39]. The islands are influenced by three water masses: coastal water (CW), resulting from the mixing of fresh water and seawater, with low salinity and an average temperature of 22°C; tropical water (TW), warm and saline, with temperatures above 18°C and salinity above 35.9 ppm; and South Atlantic central water (SACW), colder, with temperatures ranging from 6°C to 18°C and salinity from 34.5 to 35.9 ppm. In summer, SACW in the bottom layer creates a thermocline (20–50 m), intensified by northeast winds. In winter, southwest winds promote water mixing and cooling, causing the thermocline to disappear and limiting the presence of SACW to the edge of the platform. TW dominates the surface of the outer platform, while CW prevails in the inner platform [40].

On each island, four sampling points were similarly selected avoiding strong winds and rough sea in more sheltered areas, easing boat (figure 1).

### 2.2. Visual census and sampling design

**Figure 2.**
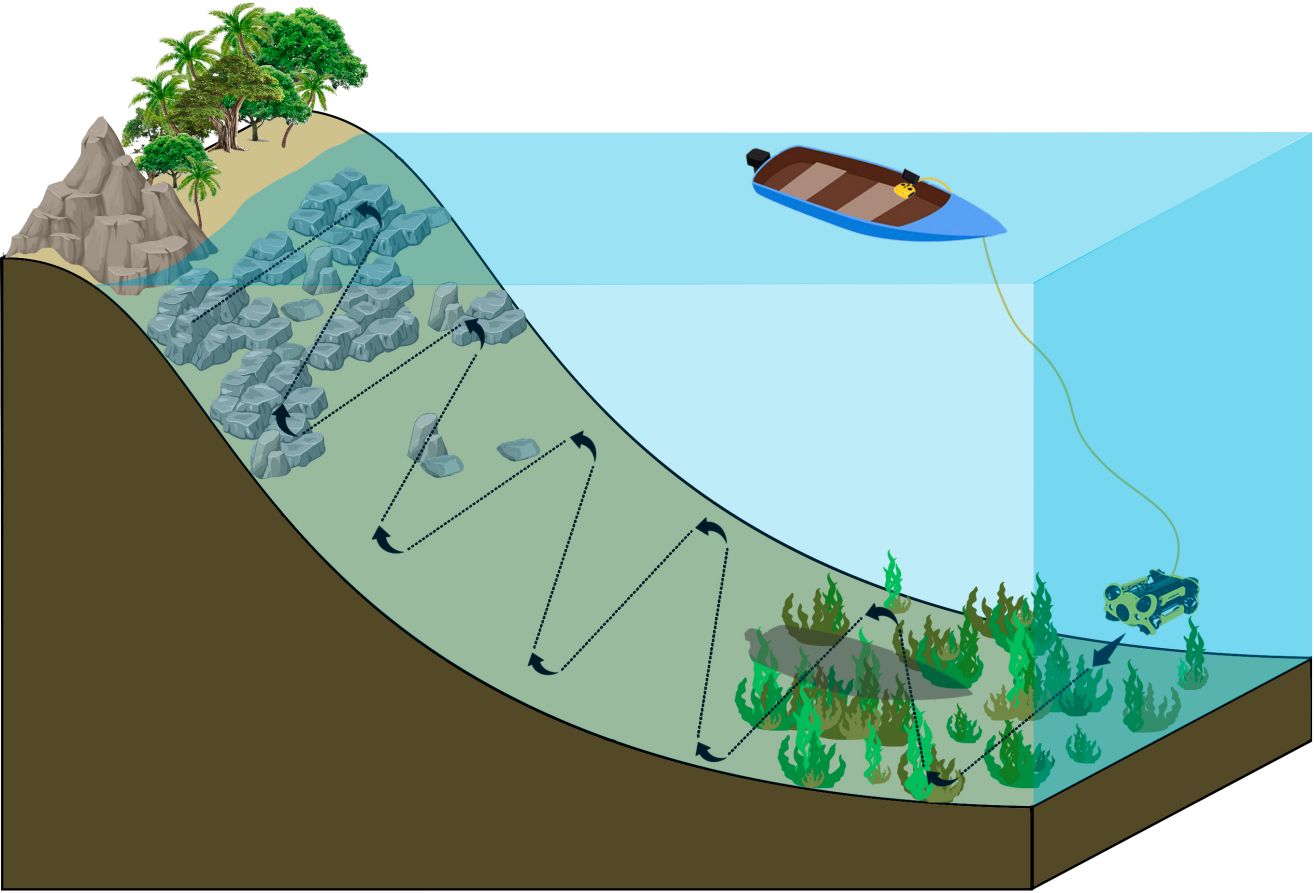
Schematic model of the operationalization with the remotely operated vehicles.

The ichthyofauna survey was conducted using the M2 CHASING mini ROV, following a visual census methodology based on Carminatto *et al.* [29] but tailored to the specific characteristics of the underwater landscape in the study areas. The operation was conducted from a boat with two trained operators: one responsible for piloting the ROV and the other for cable management. At each survey point, transects began at a distance of 30 m from the coast, covering a total of 50 m in parallel zigzag patterns toward the islands along the water column, for a fixed duration of 15 min (figure 2), at depths ranging from 2 to 7 m.

Field collections took place during the climatic cycle of 2022, considering three campaigns in each season, one per month (*summer*: January to March; *autumn:* April to June; *winter:* July to September and *spring:* October to December). In each campaign and on each island, four points were sampled during the daytime and repeated during the night time. In total, the points were sampled 192 times (48 per climatic season (four levels), 96 per island (two levels), 96 per diurnal period (two levels) and 48 per point (four levels)), with a total of 2880 min of footage.

### 2.3. Analysis of videos: fish assemblage and abiotic variables

The videos were edited using the iMovie^®^ program [41] to enhance quality in order to assist the subsequent visual fish identification in the laboratory. In order to reduce the risk of duplicate individual counts, every video was analysed by a single observer [29] and the adoption of MaxN, which is the maximum number of individuals of each species observed in a single frame in the recording [42]. Species were identified following the current literature in the field. Proper sensors attached to the ROV read temperature and depth.

### 2.4. Data analysis

For the categorization of schooling and non-schooling species, we followed Quimbayo *et al.* [43] regarding group size. Species forming large, medium or small groups were classified as schooling, while solitary or pair-living species were classified as non-schooling (electronic supplementary material, table S1). To identify conservation target species, we consulted the International Union for Conservation of Nature (IUCN) Red List [44], the Risk of Extinction Assessment System for Brazil’s Diversity—SALVE [45] and the State of São Paulo Decree No. 63853, dated 27 November 2018 [46]. For the categorization of fishing target species, we referred to Rotundo [47].

### 2.5. Statistical analyses

#### 2.5.1. Generalized linear model

In order to understand which spatial (islands), temporal (climatic seasons and diurnal period) and abiotic (temperature and depth) factors explain the numerical abundance of reef fish, generalized linear models (GLiMs) [48] were employed. The categorical variables included the two islands (Anchieta Island State Park and Mar Virado Island), the four seasons of the year (summer, autumn, winter and spring) and the two diurnal periods (day and night); the covariates abiotic variables included depth and temperature, along with their squares (assuming that there are ideal depths and temperatures associated with higher abundances). The response variables (individual counts) were total numerical abundance, numerical abundance of schooling species and numerical abundance of non-schooling species.

To handle the probability distribution different from the normal or Gaussian distribution, as required by count data [49], the negative binomial distribution and the logarithmic link function were used for total abundance. This considered categorical variables, the two covariates and their squares, and interactions between them (except for the interaction between temperature and climatic season due to their dependent relationship). For the abundances of schooling and non-schooling species, the negative binomial distribution and the logarithmic link function were used, adjusting the models as described above.

The variable selection in the aforementioned models was carried out through likelihood ratio tests at a significance level of 5%. Parameters estimation and their respective standard errors were reported only for the final models (the ‘best’ ones, after variable selection). Additionally, pairwise comparisons (Tukey type) between levels of factors present in the selected models were reported. The final statistical models were validated using residual analyses, specifically quantile–quantile plots with simulated envelopes [50]. The residual analyses of the initial and intermediate models are in the electronic supplementary material, res1 and res2.

#### 2.5.2. Exponential family nonlinear model

To understand which spatial (islands), temporal (climatic seasons and diurnal period) and abiotic (temperature and depth) factors explain reef fish richness (*S*), we adjusted several models. Due to the unsuitability of simpler models (please check the electronic supplementary material, M1 describing in detail our failed attempts) and considering that the presence of overdispersion in the data was due to the exclusion of abundance from the model, as abundance precedes richness, it was decided to include it and fit an exponential family nonlinear model (EFNLM) evaluating different predictors (or functional relationships between richness and abundance), such as Michaelis–Menten, exponential, linear, quadratic, power, logistic, Gompertz and von Bertalanffy. The Michaelis–Menten predictor, with only two parameters, was the best fit for our data, showing the lowest Akaike information criterion (AIC) [51]. Variable selection was performed through likelihood ratio tests (at a 5% significance level), and models were chosen via AIC (with a threshold difference of two points, favouring the model with the lower AIC) [51] and validated through residual analyses, specifically quantile–quantile plots with simulated envelopes [50]. Thus, parameter estimates and their respective standard errors were reported only for the final model (the ‘best’ one after variable selection). The final EFNLM included a Poisson random component, logarithmic link function and predictor formed by combining the left-hand side of the Michaelis–Menten nonlinear equation with a linear component.

#### 2.5.3. Vector generalized linear model

To model the composition and abundance of target conservation species and target fishing species between Anchieta Island State Park and Mar Virado Island, a vector generalized linear model (VGLM) with Dirichlet-multinomial random component and logistic link function was used [52]. VGLMs can be understood as an extension of GLiMs, allowing for a vector of linear predictors corresponding to the vector of parameters of the random component. In this context, we can understand the Dirichlet-multinomial distribution as a multivariate generalization of the beta-binomial distribution [53]. For both target conservation species and target fishing species, two models were fitted, one without island effect (only intercept), assuming that the composition and abundances occur randomly independent of the islands, and one model with island effect, assuming that the islands influence species composition and abundance. These models were compared through likelihood ratio tests at a significance level of 5%. From the final models, due to potential biases in the estimates of their parameters resulting from the non-observation of some species due to the sampling method employed, not only the expected proportion of occurrence of different species was evaluated but also the odds ratio was considered, i.e. how many times more one expects to find a certain species on one island compared with another.

The analyses were performed using the R platform [54] and its packages ‘MASS’ [55], ‘VGAM’ [53], ‘lme4’ [56], ‘hnp’ [57], ‘gnm’ [58] and ‘dplyr’ [59].

## 3. Results

### 3.1. Fish assemblage

A total of 16 661 individuals, distributed across 81 species, two classes, 21 orders and 38 families, were observed in the rocky reefs of the two islands. In PEIA, 9213 individuals belonging to 69 species were recorded, while in IMV, 7448 individuals from 61 species were counted (electronic supplementary material, table S1). Twenty species (24.69%) were exclusive to PEIA, and 12 species (14.81%) were exclusive to IMV. Of the total species, 49 were recorded on both islands, resulting in a composition similarity of 60.49%.

### 3.2. Numerical abundance

The quantile–quantile plot with simulated envelope identified 7 (3.6%) of the 192 points outside the envelope, which is acceptable (electronic supplementary material, figure S1:A). The significant factors (*p* < 0.05) explaining the numerical abundance of fish were the islands, diurnal period, climatic season, depth, temperature and the interaction between islands and depth (table 1).

**Table 1.** Parameter estimates for the final model of GLM (geometric random component with logarithmic function) adjusted for numerical abundance. |*Z*|: Wald test statistic. Superdispersion parameter *k* = 0.9698 and s.e. = 0.0936. Significant values are indicated in bold.

parameter	estimate	s.e.	|*Z*|	*p*
(intercept)	3.30805	1.62947	2030	0.0423
Anchieta Island State Park	−4.15946	0.93348	−4.456	**<0.0001**
night	−1.05154	0.14886	−7.064	**<0.0001**
autumn	−0.20435	0.21558	−0.948	0.3431
spring	−0.60667	0.26251	−2.311	**0.0208**
summer	−0.31528	0.33761	−0.934	0.3503
depth	−0.55785	0.15284	−3.650	**0.0002**
temperature	0.18111	0.06266	2890	**0.0038**
Anchieta Island State Park : depth	0.87083	0.18495	4708	**<0.0001**

**Figure 3.**
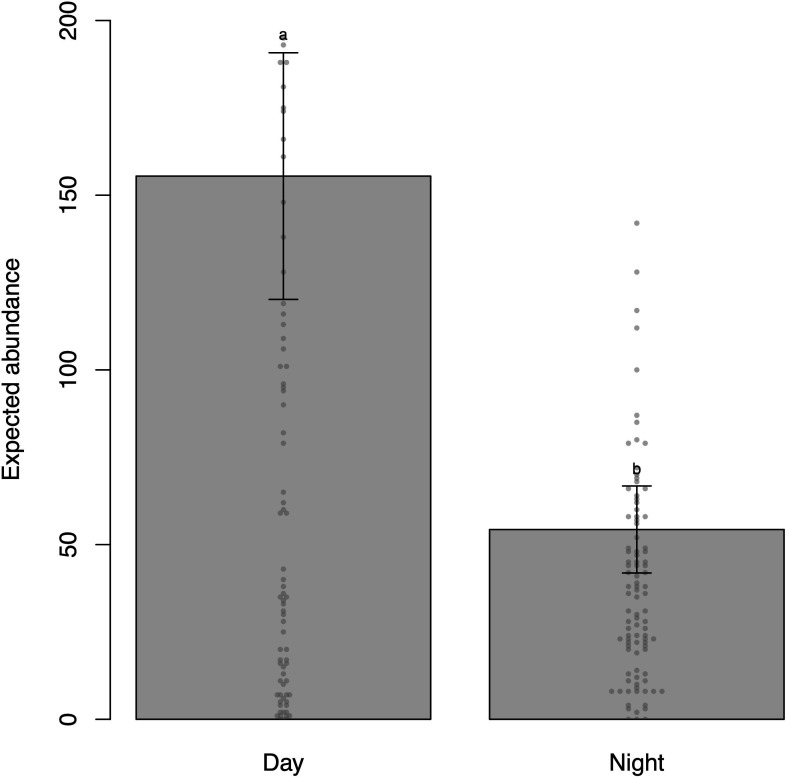
Predicted numerical abundances according to diurnal period (day and night) and their corresponding standard errors (bars). The points represent observed data. Levels marked with the same letters do not show statistically significant difference (*p* ≥ 0.05), while different letters denote significant differences (*p* < 0.05) between levels. Base scenario for plot scale (model prediction): temperature = 25°C (observed median), depth = 5 m (observed median), climatic season: winter and location: Mar Virado Island. Please note that there is no loss in interpreting the direct comparisons between levels of a factor (e.g. day and night of diurnal period) for fixed values of the other covariables when the factor under investigation is not involved in statistically significant interactions.

The diurnal period was statistically different, implying higher numerical abundance during the day compared with the night (*p* < 0.001) (table 1; figure 3).

**Figure 4.**
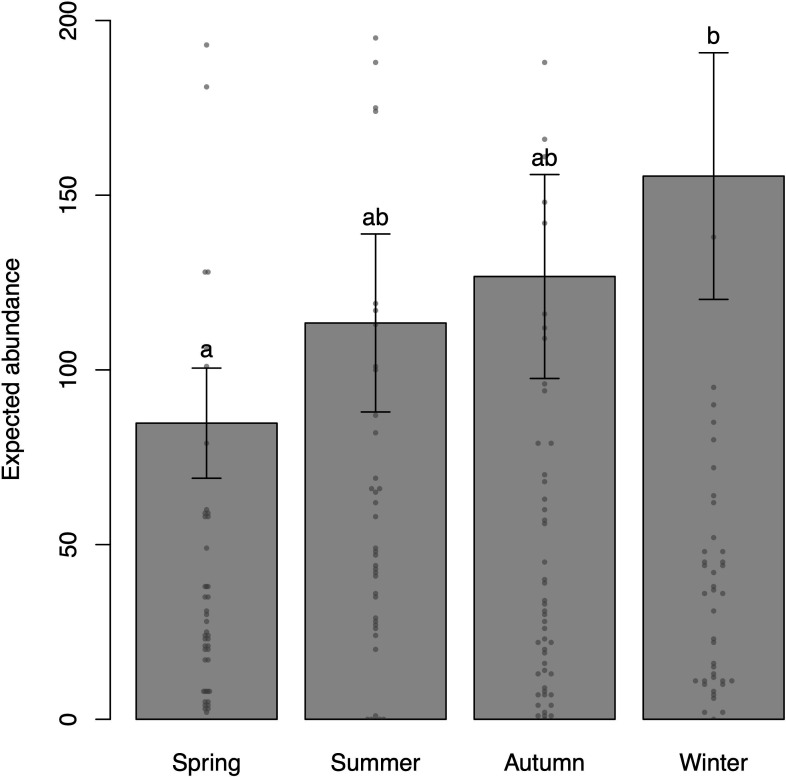
Predicted numerical abundances according to climatic season (spring, summer, autumn and winter) and their corresponding standard errors (bars). The points stand for observed data (values above 200 individuals were omitted from this plot). Levels marked with the same letters do not show statistically significant difference (*p* ≥ 0.05), while different letters denote significant differences (*p* < 0.05) between levels. Base scenario for plot scale (model prediction): temperature = 25°C (observed median), depth = 5 m (observed median), diurnal period: day and location: Mar Virado Island. Please note that there is no loss in interpreting the direct comparisons between levels of a factor (e.g. spring and fall or summer and winter of climatic season) for fixed values of the other covariables when the factor under investigation is not involved in statistically significant interactions.

The climatic season influenced abundance and showed a statistically significant difference between winter and spring (*p* = 0.0208), with higher numerical abundance observed during winter (table 1; figure 4). However, this significant difference is only detectable in the GLiM due to the robustness of the Wald *Z*-test, and it is not observable when comparing the variables pairwise using the Tukey-type test (electronic supplementary material, table S2).

**Figure 5.**
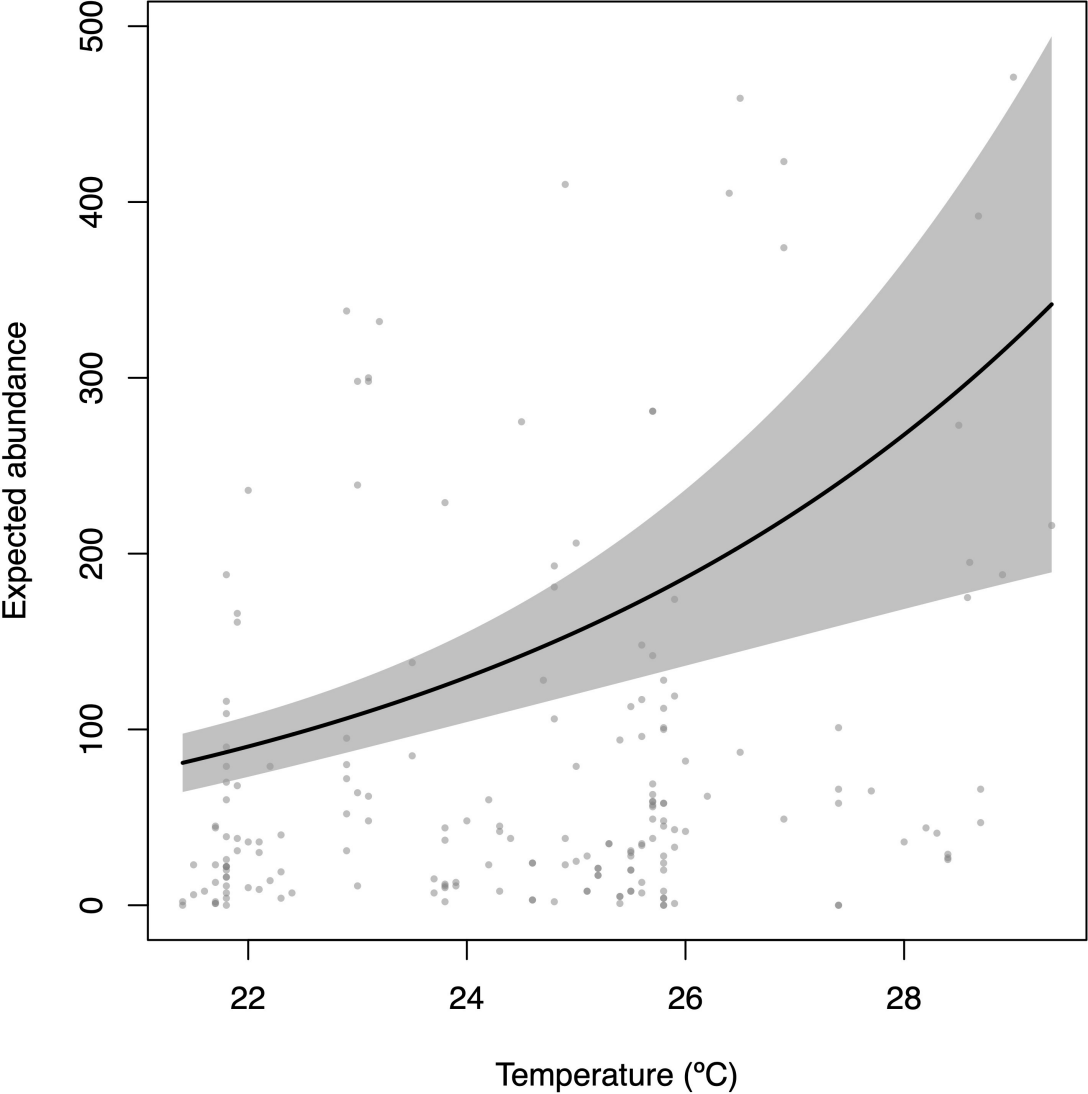
Predicted abundances according to variation in temperature (the points represent observed data). Base scenario for plot scale (model prediction): depth = 5 m (observed median), diurnal period: day, climatic season: winter and location: Mar Virado Island. Please note that there is no loss in interpreting the behaviour of the curve for fixed values of the other covariables when the covariable under investigation is not involved in statistically significant interactions.

The temperature on both islands exhibited a statistically significant difference in the model (*p* = 0.0038), indicating a positive correlation with numerical abundance and suggesting that higher temperatures are positively associated with increased fish abundance (table 1; figure 5).

**Figure 6.**
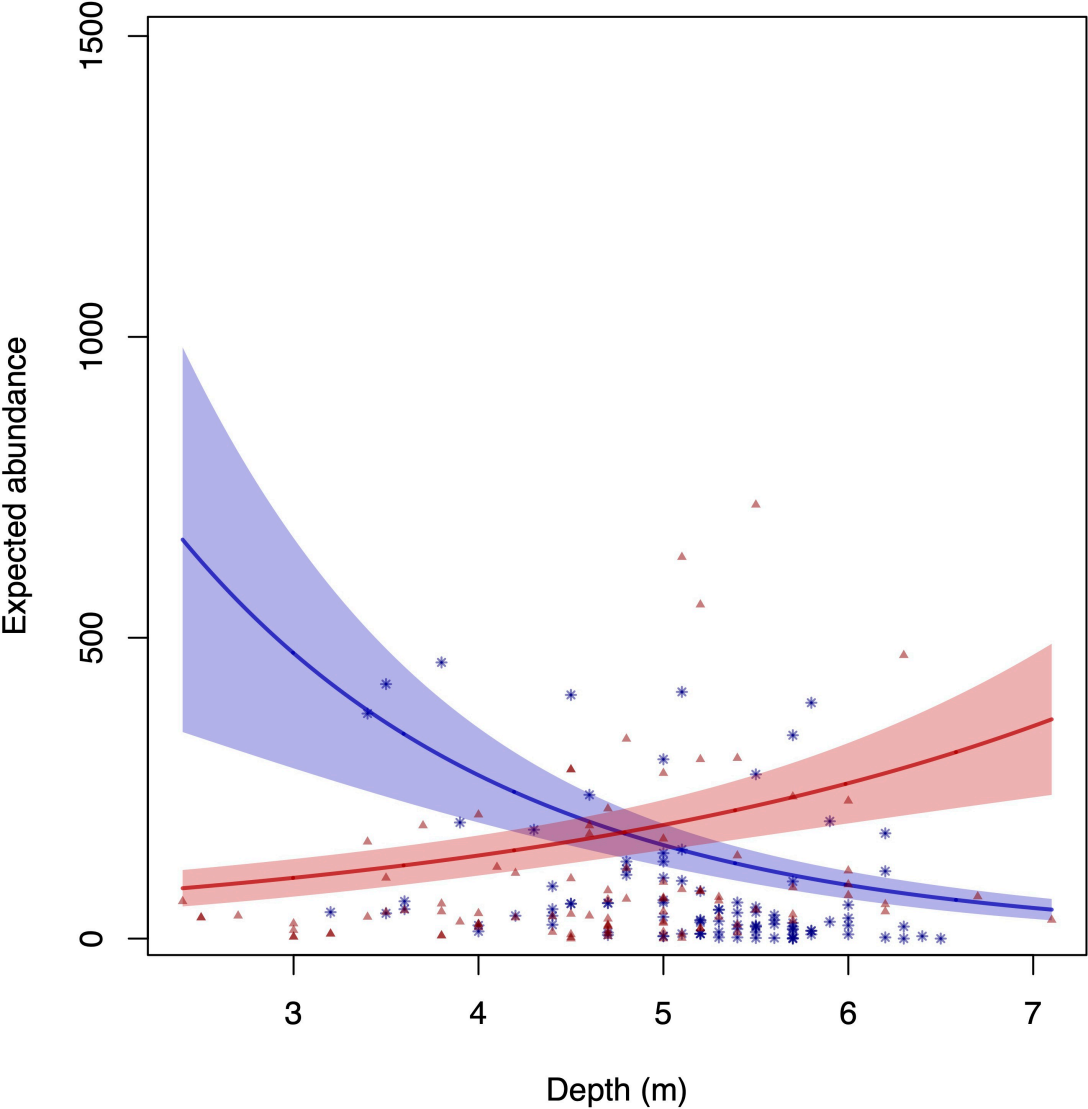
Graph of the interaction between islands and depth for numerical abundance. The blue line represents Mar Virado Island and the red line represents Anchieta Island State Park. Asterisks denote raw data points for Mar Virado Island and triangles represent raw data points for Anchieta Island State Park. Base scenario for plot scale (model prediction): temperature = 25°C (observed median), diurnal period: day, climatic season: winter and location: Mar Virado Island. Please note that there is no loss in interpreting the behaviour of the curve for fixed values of the other covariables when the covariable under investigation is not involved in statistically significant interactions.

Although numerical abundance differed between the islands and depths individually, the significant interaction observed between these factors (*p* < 0.05) implies higher numerical abundance at greater depths in Anchieta Island State Park (*p* < 0.0001), and interestingly, higher numerical abundance at shallower depths in Mar Virado Island (table 1; figure 6).

### 3.3. Numerical abundance of schooling and non-schooling species

Of the 81 species observed, 61.7% (*n* = 50) are schooling species and 38.3% (*n* = 31) are non-schooling species. The final models of the selected GLiMs for numerical abundance of schooling and solitary species were those with a negative binomial random component and a logarithmic link function, with the following overdispersion parameters *k* = 0.9195 and 0.879 and s.e.: 0.0907 and 0.103, respectively (electronic supplementary material, table S3). Quantile–quantile plots with simulated envelopes identified 16 (8.33%) and 29 (15.10%) of the 192 points outside the envelopes, respectively (electronic supplementary material, figure S1B and C).

The significant factors (*p* < 0.05) explaining the numerical abundance of schooling and non-schooling species were the same as those for total numerical abundance (islands, diurnal period, climatic season, depth, temperature and the interaction between islands and depth), but with the addition of specific factor interactions for each behaviour (electronic supplementary material, table S3). For schooling species, in addition to the significance of the interaction between island and depth, other significant factors included interactions between diurnal period and climatic season, implying higher schooling activity at night during autumn (*p* = 0.0185), and the interaction between depth and temperature, suggesting schooling in deeper and warmer waters (*p* = 0.0330) (electronic supplementary material, table S3). For non-schooling species, in addition to the significance of the interaction between island and depth, there was an interaction between diurnal period and temperature, indicating lower abundance of solitary species at night when the temperature decreases (*p* = 0.026), and the significance of the climatic season factor, indicating differences between winter and autumn (*p* = 0.0004), with higher numerical abundance of solitary species during winter (electronic supplementary material, table S3).

### 3.4. Richness

The Michaelis–Menten predictor with only two parameters was the best fit for our data, showing the lowest AIC. The quantile–quantile plot can be observed in electronic supplementary material, figure S1D. The significant parameters of the EFNLM explaining richness were the diurnal period, the interaction between islands and the diurnal period and the *S*_max_ and *B* parameters of the Michaelis–Menten model (table 2).

**Table 2.** Estimates of parameters for the final model of EFNLM (nonlinear predictor of Michaelis–Menten and Poisson random component with logarithmic function) adjusted to richness. |*Z*|: Wald test statistic. AIC: 880.22. *S*_max_: total number of species in the community and *B*: sample effort required to detect 50% of *S*_max_. Significant values are indicated in bold.

parameter	estimate	s.e.	|*Z*|	*p*
(intercept)	−0.1802	0.3113	−0.579	0.5626
Anchieta Island State Park	−0.0648	0.0665	−0.974	0.3300
night	−0.2500	0.0786	−3.179	**0.0014**
Anchieta Island State Park: night	0.2496	0.1009	2474	**0.0133**
*S*_max_	2.8898	0.3016	9581	**<0.0001**
*B*	6.6199	1.5347	4313	**<0.0001**

**Figure 7.**
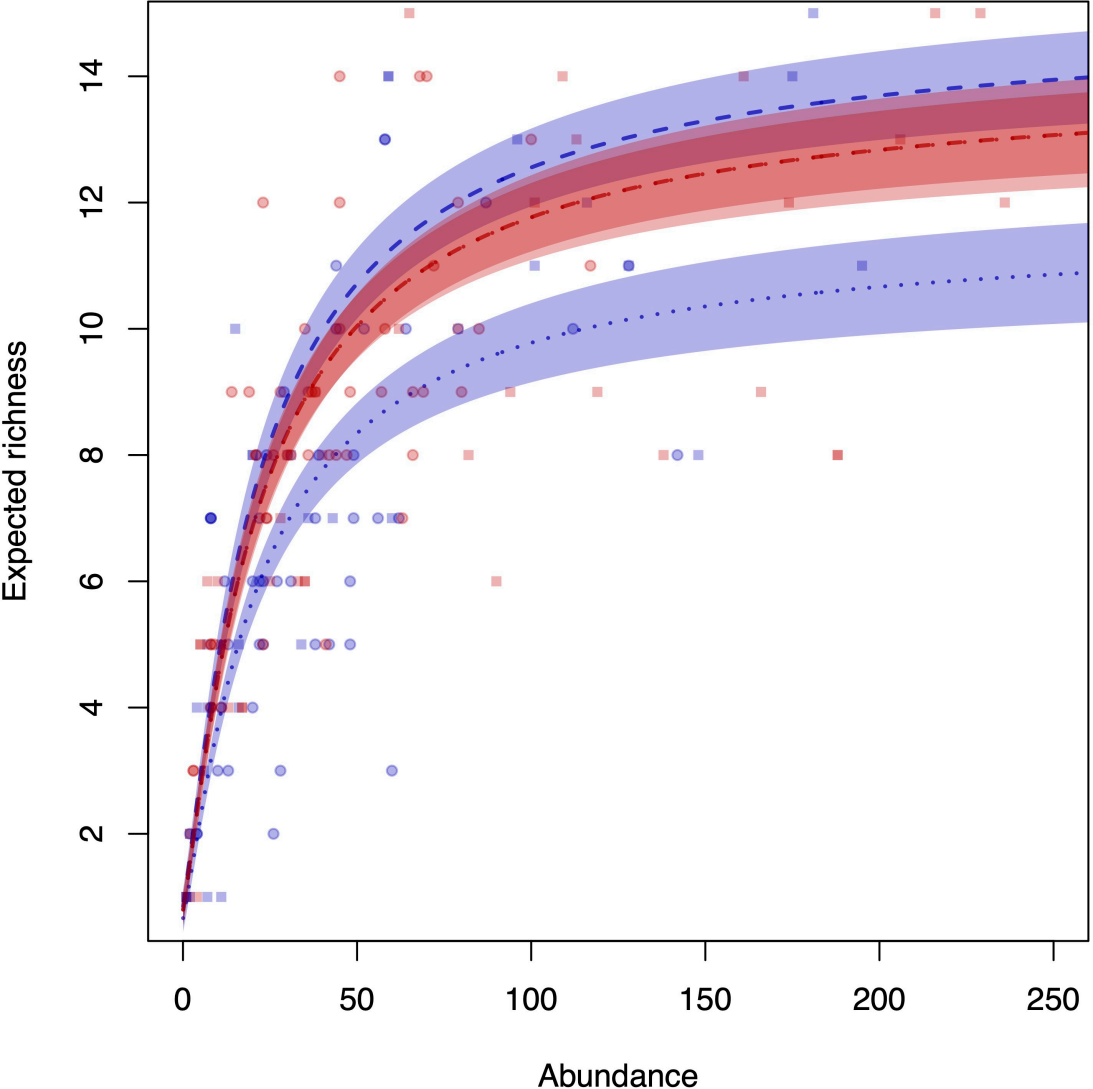
Graph of expected richness with the Michaelis–Menten model and the interaction between islands and diurnal period. The blue line represents Mar Virado Island and the red line represents Anchieta Island State Park. The dashed line represents the day and the dotted line represents the night. Squares represent raw data for the day and circles represent raw data for the night.

The Michaelis–Menten model (*p* < 0.0001) proved to be a good predictor for estimating richness on both islands, revealing that the species accumulation curves for both Anchieta Island State Park and Mar Virado Island are stable and asymptotic, meaning they approach a limit as more samples are collected. This suggests that if sampling continued, the addition of new species would be minimal, as the total number of species is finite on both islands (i.e. the Michaelis–Menten curve has an asymptote/saturation). This analysis reinforces that the sampling effort was sufficient to record the fish richness of the two islands (table 2; figure 7). The significant interaction between the islands and the diurnal period highlights that the diurnal period influenced only Mar Virado Island, where greater richness is observed during the day compared with the night, while at Anchieta Island State Park there is no significant difference in richness between day and night (table 2; figure 7).

### 3.5. Composition and proportion of occurrence of conservation target species and fishing target species between the islands

Considering the three conservation status, lists ([44–46,]; 17.3% (*n* = 14)) of the species were classified as being under some degree of threat and are considered conservation targets (table 3).

**Table 3.** Conservation status of species according to the IUCN red list [44], the Risk of Extinction Assessment System for Brazilian Diversity—SALVE [45] and the Decree of the State of São Paulo No. 63853, dated 27 November 2018 [46], classified as: least concern (LC), vulnerable (VU), near threatened (NT), endangered (EN), critically endangered (CR), data deficient (DD) and not assessed (NA).

species	conservation status		
	IUCN	SALVE	SMA
*Aetobatus narinari*	EN	DD	NT
*Centropomus undecimalis*	LC	DD	NT
*Dasyatis hypostigma*	EN	DD	NA
*Epinephelus marginatus*	VU	VU	EN
*Gymnura altavela*	EN	CR	EN
*Hyporthodus niveatus*	VU	VU	EN
*Lutjanus analis*	NT	NT	NA
*Lutjanus synagris*	NT	NT	DD
*Orthopristis rubra*	LC	LC	NT
*Pomacanthus paru*	LC	DD	NT
*Pseudobatos percellens*	EN	VU	EN
*Selene vomer*	LC	LC	NT
*Sparisoma axillare*	DD	VU	EN
*Sparisoma frondosum*	DD	VU	EN

When comparing models with and without the island effect, it is observed that there is no island effect on the composition and abundance of conservation target species (table 4).

**Table 4.** Generalized linear vector models with Dirichlet-multinomial random component adjusted to the composition and proportion of expected occurrence of conservation target species with and without the island effect. d.f.: degrees of freedom.

parameters	GL residual	residual likelihood function	d.f.	2 × log likelihood function	*p*
without island effect	2405	−1777	—	—	—
with island effect	2393	−84517	12	−165481	—

Out of the 81 species observed, 32.1% (*n* = 26) are targeted by fishing and 67.9% (*n* = 55) have no commercial value. When comparing models with and without the island effect, it is observed that there is an influence of the island on the composition and proportion of occurrence of fishing target species (*p* < 0.0001) (table 5). Among them, four species have their proportions altered from one island to another, namely: *Anisotremus surinamensis, Caranx latus, Stephanolepis hispida* and *Epinephelus marginatus* (*p* < 0.0001) (electronic supplementary material, table S4).

When comparing the ratio between the proportions of each species on each island, it is observed that there is 3.3 times more *C. latus* and 4.3 times more *E. marginatus* in Anchieta Island State Park, and 2.4 times more *A. surinamensis* and 4.1 times more *S. hispida* on Mar Virado Island (table 6).

**Table 5.** Generalized linear vector models with Dirichlet-multinomial random component (likelihood function) adjusted to the composition and proportion of expected occurrence of fishing target species with and without the island effect. d.f.: degrees of freedom.

parameters	GL residual	residual likelihood function	d.f.	2 × log likelihood function	*p*
without island effect	4995	−4939.2	—	—	—
with island effect	4969	−4902.6	26	73 355	**<0.0001**

**Table 6.** Expected proportions (*P*) and ratio (*R*) of each species on each island based on the generalized linear vector model with Dirichlet-multinomial random component (electronic supplementary material, table S4).

islands	*Anisotremus surinamensis*		*Caranx latus*		*Stephanolepis hispida*		*Epinephelus marginatus*	
	*P*	*R*	*P*	*R*	*P*	*R*	*P*	*R*
Anchieta Island State Park	0.0069	0.41	0.0209	3.26	0.0018	0.24	0.0070	4.33
Mar Virado Island	0.0169	2.42	0.0064	0.31	0.0076	4.17	0.0016	0.23

## 4. Discussion

The Michaelis–Menten predictor was used for richness data, with its asymptote interpreted in this context as an estimate of richness. Based on the results, the estimator proved to be a good predictor for estimating richness on both islands, indicating that the sampling effort employed was sufficient to record richness in Anchieta Island State Park and Mar Virado Island (table 2; figure 7). The use of this method in ecology is classic, and according to Magurran [60], the Michaelis–Menten model still serves as a practical rule for the sampling stopping criterion: sampling continues until the collector’s curve crosses the curve generated by the model. Petrere & Butturi-Gomes [61] show that it is impossible to provide confidence intervals for *S*, the estimated number of species, as there is no independence between *S* and the accumulated collection effort (*f*), even if the data are independently collected in the field. This is an intrinsic problem of the model that randomization cannot remedy.

The total fish abundance varies between diurnal periods, with higher abundance observed during the day (table 1; figure 3). This result is expected, as it is known that the daily cycle influences the structure and composition of reef fish communities [8]. Diurnal changes in fish communities have been investigated in different environments: in rivers, lower abundance was observed at dusk due to the presence of large carnivorous fish at night [62–64]; in mangroves, nocturnal fish abundance was affected by predation risk and food availability [65,66]. In estuaries, higher night-time catch rates were associated with increased activity of planktivorous and carnivorous fish migrating from mangroves to adjacent habitats to forage, especially after sunset [67]. In reefs, nocturnal fish assemblages were less diverse and less abundant than diurnal ones [8,68], and in MPAs, fish community structure varies between day and night, with greater nocturnal dispersion and higher fish abundance in areas closed to fishing [9]. Particularly in Anchieta Island State Park, Furia [69], who studied the spatio-temporal variation in the composition and structure of ichthyofauna in Enseada das Palmas, contrary to our result, recorded higher abundance and richness at night. Previous research has identified that differences in fish assemblage distributions throughout the day are influenced by shelter availability [70], prey abundance [13,14], predation risk [66,71], behavioural changes, such as daily activity [72], and schooling behaviour [73].

When analysing schooling species, we found that the interaction between diurnal period and climatic season was a significant factor, indicating that schooling formations were more prominent at night and during autumn (electronic supplementary material, table S3). This can be explained by the high number of juveniles of *Anchoa* spp.; species of this genus are schooling species and approach the coast during colder months [74]. As for non-schooling species, the significant interaction between diurnal period and temperature led to lower abundance during the night and at lower temperatures (electronic supplementary material, table S3). Several studies have shown a decrease in schooling formation as night falls. Fréon *et al.* [73] emphasized that at dawn, fish tend to aggregate rapidly, forming schools, while at dusk, school dispersion is slower. The author discusses that diurnal variations in fish schooling formation are related to the reach of their vision, which is greater during the day due to natural light. At dusk, fish swim to feed or protect themselves, resulting in a slow expansion of the school to areas adjacent to the reefs where space is available. From a behavioural perspective, dusk dispersion is mainly passive, while aggregation before dawn is primarily active.

Seasonal variation influenced the total numerical abundance (table 1; figure 4) and the abundance of non-schooling species (electronic supplementary material, table S3), with higher abundance observed during winter, probably linked to the high numerical abundance of *Abudefduf saxatilis, Stegastes fuscus, Haemulon aurolineatum* and *Harengula clupeola*. Additionally, during winter, southwest winds promote water mixing and cooling, causing the disappearance of the thermocline observed in summer and limiting the presence of SACW to the edge of the platform. Warm and saline TW dominates the surface of the outer platform, while low-salinity CW, with an average temperature, prevails in the inner platform [40], favouring species abundance. The structure of reef fish communities is influenced by both spatial habitat characteristics and seasonal variation [29,75]. Seasonal changes, such as variations in water temperature, ocean current patterns, reproduction cycles and food availability, impact the structure of reef fish communities [16,76,77]. For example, certain species migrate seasonally to specific areas for feeding or reproduction [78,79]. Seasonal changes in temperature also affect the distribution and habitat utilization of roaming herbivorous fish; in addition, reefs are subject to influences from upwelling, resulting in variations in environmental conditions, leading to significant changes in the composition and abundance of benthic algae [77,80]. A study in the southern Red Sea showed that herbivores tend to explore deeper reef zones in the summer, while in other seasons, they aggregate in shallower reef zones [81]. Vaughan *et al.* [16] assessed seasonal variation in reef fish assemblages at the southern tip of the Persian/Arabian Gulf and identified that fish abundance is generally higher in summer than in winter, with significant differences when evaluating compositional similarity between the two seasons.

Temperature showed statistical significance in the model, indicating a positive correlation with total numerical abundance, suggesting that higher temperatures are positively associated with greater fish abundance, despite higher variability in the data (table 1; figure 5). For schooling species, the interaction between depth and temperature indicated aggregation in deeper and warmer waters (electronic supplementary material, table S3). These results may be linked to the oscillation of the subtropical convergence between the warm waters of the Brazil Current and the cold waters of the Malvinas Current in southern Brazil. The study area features upwelling zones that, during the summer, allow the SACW to penetrate the shelf, reaching coastal areas with a thermocline of 10–15 m. During the winter, the SACW retracts and TW and CW fill this space [40]. These frequent influxes of colder waters, combined with the relatively wide depth range at Anchieta Island State Park (table 1; figure 6), could explain the presence of several reef fish species with subtropical and temperate affinities, as several researchers have already suggested [82–87].

Additionally, this pattern may be associated with the latitudinal gradient, where tropical marine fish communities are significantly more diverse than those found at higher latitudes [88,89]. Several explanations for this latitudinal diversity gradient propose that warm reef environments act as evolutionary ‘hotspots’ for species formation [90–93]. Moreover, rising temperatures due to climate change have been altering the diversity, structure and functioning of fish communities, leading to increased metabolic stress, shifts in growth and reproduction rates and changes in species geographical distributions [94,95]. Over time (2008−2020), tropical species have become gradually more abundant, while temperate species have declined, reflecting an initial shift in fish composition within this transitional zone of the southwestern Atlantic [96].

Furthermore, although numerical abundance differed between islands and depths separately, the significant interaction observed between these factors implies higher total numerical abundance (table 1) and higher abundance of schooling and non-schooling species (electronic supplementary material, table S3) at greater depths in Anchieta Island State Park and higher abundance at shallower depths in Mar Virado Island. This result may be related to the structural complexity of the habitat in Anchieta Island State Park, where increased habitat complexity provides more shelter, feeding, reproduction and recruitment sites, thus increasing the number of individuals and species [29,97–100]. Teixeira-Neves *et al.* [101] observed greater fish diversity in deeper locations compared with shallow ones. They associated this greater diversity with deep locations situated between consolidated substrate and sandy bottoms. This transition zone favours increased species richness as it provides a suitable environment for species to move for foraging activities and camouflage in the sand. In contrast, shallow locations are characterized only by consolidated substrate, thus offering less habitat diversity for fish. Mendonça-Neto *et al.* [102] also found that species richness increases from shallow to interface areas in a tropical region along the southeast coast of Brazil.

In the richness model, the significant interaction between the islands and diurnal period highlights that the diurnal period only varied in Mar Virado Island, where higher richness was observed during the day. In contrast, in Anchieta Island State Park, no significant difference in richness was found between day and night (table 2; figure 7). In Mar Virado Island, diurnal variation is probably attributed to changes in trophic relationships, such as the vertical distribution of prey and behavioural changes in response to increased nocturnal predation [13,14]. However, this result was expected for Anchieta Island State Park, which is a no-take area, and it is anticipated that no-take MPAs typically protect species targeted by fishermen who are often the main predators in the ecosystem [103]. Thus, one would expect these areas to be subject to higher predation pressures, particularly from nocturnal species. This pattern was recorded in the Houtman Islands in Western Australia, where despite the reduction at night, higher numbers of individuals were still observed within the no-take areas than in open areas during nocturnal surveys [1].

When comparing models with and without the island effect, it is observed that there was no island effect on the composition and abundance of conservation target species (table 4). In contrast, for target fishing species, the island influences the composition and proportion of their occurrence (table 5). For example, when comparing the ratio between the proportions of each species on each island, there is an occurrence of 3.26 times more *C. latus* and 4.33 times more *E. marginatus* in Anchieta Island State Park and 2.42 times more *A. surinamensis* and 4.17 times more *S. hispida* at Mar Virado Island (table 6). Some studies show that no-take MPAs have been effective in restoring populations of target species, such as piscivores [104], and effective in increasing biodiversity, especially for exploited fish species [3]. In addition to the fishing protection polygon in Anchieta Island State Park, the occurrence of predators *C. latus* and *E. marginatus* is due to the structural complexity of rocky shores [29,82,101,105,106], as the depth on this island positively influences the numerical abundance of schooling and non-schooling species. Furthermore, these locations are suitable for predators like serranids in search of prey [101]. On the other hand, at Mar Virado Island, the higher occurrence of species *S. hispida* and *A. surinamensis* may be associated with the presence of large banks of algae and marine invertebrates. Boaden & Kingsford [107] observed in the Great Barrier Reef Marine Park in Australia that open-to-fishing reefs had lower densities of piscivores and higher densities of prey and herbivorous fish compared with closed marine reserves.

Predatory fishing and by-catch are the main stress factors affecting over 17% of threatened species recorded in the studied islands, primarily mesopredators (medium to large-sized carnivores) such as groupers (*E. marginatus and Mycteroperca acutirostris*), snappers and lutjanids (genus *Lutjanus*), as well as large herbivores like *Sparisoma axillare* and parrotfish (genus *Sparissoma*) [6,25,101,108]. Fishing has targeted small-sized fish occupying lower trophic levels, signalling unsustainable fishing. In 2015 alone, approximately 50 000 tons of commercially valuable fish were caught. The lack of management and oversight of fishing activities has led to the decline of various fish populations, particularly since the 2000s, when there was a peak in the catches of these species [25].

The elasmobranch species recorded (4.9%) are classified as endangered in some of the lists [44–46]; *Pseudobatos percellens, Dasyatis hypostigma, Gymnura altavela* and *Aetobatus narinari*, and their population status is more critical than that of the actinopterygians. With reduced populations, elasmobranchs are currently mainly caught as by-catch [109] and, therefore, deserve specific mitigation measures aimed at fisheries targeting other resources (especially longlines, gillnets and trawl nets) [83]. Among the actinopterygians, groupers (epinephelids), classified as threatened in all lists, are protogynous hermaphrodites with slow growth, long life and late maturity [110–112], making them highly vulnerable to fishing.

## 5. Conclusion

The study revealed that fish abundance varies significantly between daytime periods and climatic seasons, being higher during the day, in winter and areas with higher temperatures. At night, especially in autumn, changes in the behaviour of schooling species were observed. Seasonal variations, including temperature changes and the oceanographic characteristics of the study area, influenced fish abundance and species composition, favouring tropical and subtropical species. Depth influenced the islands distinctly: at Anchieta Island State Park, abundance was greater in deeper areas, while at Mar Virado Island, it was more abundant in shallower areas due to the structural complexity of the habitat. Diurnal variation in species richness was significant only at Mar Virado Island, possibly in response to increased nocturnal predation, as this is an area open to fishing. For target fishing species, the island exerts a significant influence, affecting the composition and abundance of species, which highlights the importance of protected areas. The study provides valuable insights into the dynamics of reef fish communities, highlighting the essential role of spatial–temporal and anthropogenic factors in their structure and composition. These findings can guide conservation and management strategies aimed at preserving marine diversity in MPAs).

## Data Availability

Data is available from [113]. Supplementary material is available online [114].
